# The Psychological Dimension of Diabetes: Exploring the Role of Self-Esteem, Anxiety, and Depression

**DOI:** 10.1192/j.eurpsy.2026.11028

**Published:** 2026-07-31

**Authors:** S. Antonopoulou, A. Zartaloudi, S. Parissopoulos, E. Vlachou

**Affiliations:** 1 Department of Nursing, MSc “Education and Care in Diabetes Mellitus”, University of West Attica; 2 Department of Nursing, Associate Professor, University of West Attica; 3 Department of Nursing, Assistant Professor, University of West Attica; 4 Department of Nursing, Professor, University of West Attica, Athens, Greece

## Abstract

**Introduction:**

Diabetes mellitus (DM) is a chronic metabolic condition with substantial physical and psychological complications that profoundly impact patients’ daily lives and overall quality of life. Psychological factors such as depression and anxiety are increasingly recognized as major contributors to disease progression, while self-esteem may act both as an indicator and as a potential protective factor.

**Objectives:**

The present study aimed to examine the impact of self-esteem, anxiety, and depression on the well-being of adults living with DM.

**Methods:**

A cross-sectional quantitative study was conducted with 100 adult patients with diabetes monitored at a public hospital in Attica, Greece. Data were collected using validated psychometric instruments, including the Depression Anxiety Stress Scale-21 (DASS-21), the Rosenberg Self-Esteem Scale (RSES), and the Mental Health Continuum–Short Form (MHC-SF). Sociodemographic and clinical data were also recorded and analyzed.

**Results:**

Significant negative correlations were found between depression, anxiety, stress, and psychological well-being, confirming their detrimental effect on patients’ quality of life. In contrast, higher self-esteem was positively associated with psychological, emotional, and social well-being, and negatively associated with anxiety and depression. Participants reporting comorbidities or chronic complications showed lower self-esteem and a greater psychological burden.

**Conclusions:**

The findings underscore the role of self-esteem as a potential protective factor against the psychological consequences of DM and highlight the importance of a holistic approach to care that integrates psychological support and self-image enhancement. Interventions targeting self-esteem alongside the management of anxiety and depression may substantially improve the well-being of individuals with DM, particularly in primary healthcare settings. These results may inform the development of personalized care plans and preventive strategies for psychological distress in patients with chronic illness.

**Disclosure of Interest:**

None Declared

